# Saskatchewan Movement Disorders Program

**DOI:** 10.1017/cjn.2015.13

**Published:** 2015-03

**Authors:** Ali H. Rajput, Alex Rajput

**Affiliations:** Saskatchewan Movement Disorders Program, Neurology Division, University of Saskatchewan and Saskatoon Health Region.

**Keywords:** Brain function, essential tremor, movement disorders, neuropathology, Parkinson disease

## Abstract

We review the Saskatchewan Movement Disorders Program, which started in 1968 and has had the dual goals of patient care and research. The clinics are structured to collect research-worthy data including videos, longitudinal follow-up, and autopsy studies of patients seen in the clinics. At every clinic visit, the patient is evaluated by one or both authors. A total of 25% to 30% of the deceased come to autopsy. Frozen half-brain and formalin-fixed remnants from autopsy are preserved in our laboratories. Patients not seen in our clinic are not included in research, which makes it different from brain banks. So far, 515 cases have come to autopsy. So far, there have been 17 collaborating scientific teams from Canada, the United States, Europe, and Japan. The collaborators are not charged for access to our resources. This program offers a unique opportunity to study multiple aspects of movement disorder patients seen in clinical practice.

## RÉSUMÉ

**Movement Disorders Program de la Saskatchewan.** Nous avons revu le Movement Disorders Program de la Saskatchewan établi en 1968. Son but est double, soit les soins aux patients et la recherche. Les cliniques sont structurées de telle sorte que des données de qualité soient générées à des fins de recherche, incluant des vidéos, sur le suivi à long terme et l’autopsie de patients suivis à la clinique. À chaque visite à la clinique, le patient est évalué par l’un des auteurs ou par les deux. Une autopsie est effectuée chez 25% à 30% des patients qui décèdent. Des demi-cerveaux congelés provenant d’autopsies et des tissus conservés dans la formaline sont également conservés dans nos laboratoires. Les patients qui ne sont pas suivis à notre clinique ne sont pas inclus dans notre recherche, contrairement aux banques de cerveaux. À ce jour, 515 autopsies ont été réalisées et 17 équipes de recherche du Canada, des États-Unis, d’Europe et du Japon ont collaboré avec nous. Aucun frais n’est exigé des collaborateurs pour avoir accés à nos ressources. Ce programme offre une opportunité unique d’étudier les multiples aspects des troubles du mouvement rencontrés en pratique clinique.

## Introduction and History

Ali Rajput joined the neurology faculty at the University of Saskatchewan in July 1967 and Alex Rajput joined in 2000. For the first 33 years of this history, the term “I” means Ali Rajput and the subsequent term “we” means both neurologists.

I came to the University of Saskatchewan in July 1967 on a one-year contract. In late September, I informed the department head that I was looking for a position elsewhere after my contract expired. He asked me to wait for two weeks before making the final decision as he was going to the United States and wanted to talk to me on his return. While there, he died suddenly. A new department head was appointed, and I went to inform him about my decision. The department head was highly distressed because there were not many neurologists in Canada at that time. During that meeting, I made a commitment to stay one more year in Saskatoon. During that year (1968), I met my future wife. Soon after we were married, our family circumstances changed and the decision to move was postponed.

Movement disorders were of clinical interest to me during my neurology training. Like most chronic neurological diseases, treatment options for Parkinson disease (PD) were very limited.

## Major Breakthrough

In 1960, Ehringer and Hornykiewicz reported marked striatal dopamine loss in parkinsonism.^1^ One year later, Birkmayer and Hornykiewicz reported 20 PD patients who improved in a dose-dependent fashion on intravenous levodopa (LD).^2^ Those two articles were published in German and the intravenous use of the drug had limitations for ongoing treatment of PD. A brief report of oral LD producing improvement in PD was presented by Dr. André Barbeau at the International Congress of Neurology in Rome in 1961,^2^ but received little attention. In 1967, Cotzias et al^3^ reported dramatic improvement of PD motor symptoms on a large dose of oral D-L dopa. Most cases received 9 g or more—up to 16 g/day.^3^ Soon LD became available to some experts, but it was expensive. There were many seriously disabled parkinsonian patients in every community who needed urgent treatment. There was no dose-finding study of LD, and the neurologists used the dose that they felt comfortable with.

### Impetus for the University of Saskatchewan Movement Disorders Program

In early 1968, Dr. A. Barbeau visited the University of Saskatchewan and presented his own observations and the work of Cotzias’s group. He informed us that he was organizing a multicenter LD drug trial in Canada, but that Saskatoon was not included. At that time, the University of Saskatchewan had three full-time neurologists—the third largest number of academic neurologists at a Canadian university. I asked my senior colleagues why we were not considered for the LD trial. I felt that our patients deserved the best available treatment as much as anyone else in Canada; they suggested that I consider providing that treatment.

### Start of Special Clinics and Research

In 1968, LD was not approved for general use by Canadian physicians; therefore, I needed approval from Health Canada to use the drug. Health Canada asked for evidence that I would also be pursuing research. I produced a research protocol and the permission to use LD was granted. The requirement by Health Canada made it necessary for my special clinics and research be carried out simultaneously. The clinics were started in Saskatoon in 1968, and because of patient requests we started similar monthly clinics in Regina in 1999.

Initially I had to import LD powder from the United States. The Royal University Hospital Pharmacy prepared 500-mg LD capsules (without charge) and the medication was sold to patients at cost. Several North American neurologists were already using the drug and I was in the second wave of specialists to use LD. In the early years, we admitted patients to the hospital to start LD treatment. During hospitalization, other staff—nurses, occupational therapists, physiotherapists, psychologists, and physicians-in-training—became interested in the new treatment and helped perform detailed patient evaluations.

Our movement disorders clinics in the 1960s and early 1970s included PD cases because many of those patients needed urgent treatment; however, soon I started seeing patients with other movement disorders. It was anecdotally known that essential tremor (ET) patients improved on alcohol but it had never been studied systematically; the effect of alcohol on action tremor in other disorders was also unknown. With the help of occupational therapists, I conducted a clinical pharmacology study. We observed that a small quantity of oral alcohol improved action tremor in the majority of ET cases as well as in other disorders.^4^ ^,^ ^5^

### Basic Scientists and Clinician Scientist Teams

The three essential ingredients to pursue research are: (1) appropriate topics to study; (2) material (resources); and (3) proper methods. Basic scientists are trained in highly specialized methodology that they use to answer scientific questions. Clinicians have many questions like: What is it? What caused it? How does it evolve? What is the best treatment? These different questions require different methods to provide answers. Teams consisting of clinicians and basic scientists are therefore an ideal combination for research. My research was based on many questions requiring special tools to provide answers; thus, I needed expert collaborators using specific methodology but had to provide them with adequate material. With time, many questions were also raised by the collaborators, which in turn needed our resource to answer.

## Start of Low-Dose LD Therapy

**Figure 1:**
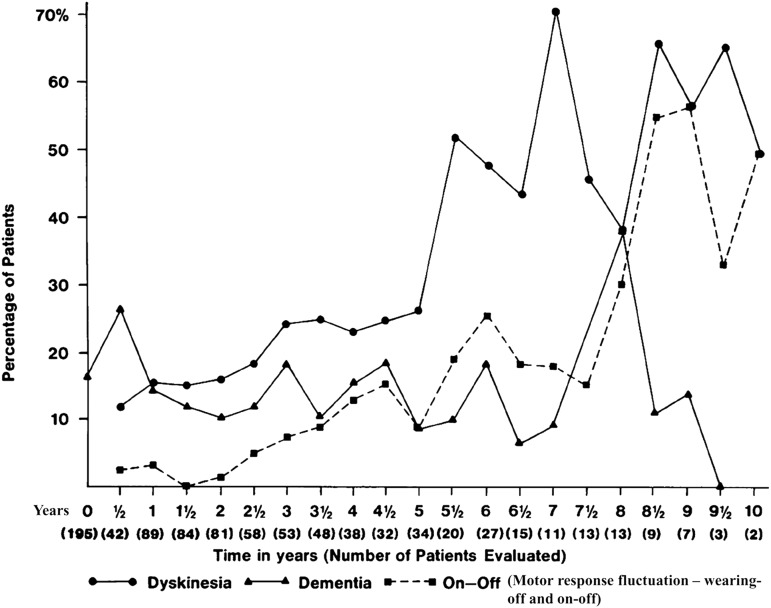
Adapted from Rajput et al, Neurology 1984.^8^

LD had created much publicity and optimism in physicians, patients, and families. Before the start of the Movement Disorder Clinic Saskatchewan (MDCS), some Saskatchewan patients had travelled to other provinces and the United States for treatment with LD. Expenses of the physician and hospital services outside the province were the responsibility of patients, unless they sought prior written approval from the provincial government. When patients that had their LD treatment initiated outside Canada reapplied for an out-of-province return visit, the government directed them to my clinic. Thus I saw patients who were already receiving LD. Based on observations in those cases and my own early experience, I recognized that the large doses of LD used by most neurologists at the time produced early and sometimes disabling dyskinesias. I pondered the benefit and adverse effects profile and concluded that in some PD cases, the quality of life on LD was not much better than the untreated disease. My observation on dyskinesias was confirmed by others at a symposium organized by Barbeau in Val David, Quebec, in 1969. Therefore, on my own, I decided to use a lower dose of LD (up to 3 g, the equivalent 600 mg levodopa/carbidopa).^6^ We hospitalized the patients for slow titration and monitored the side effects. Our patients had slower improvement, but at the end of several months they were doing as well as those on the higher LD dose and had fewer side effects. One adverse effect of LD was postural hypotension, which was the topic of an early clinical pathological study.^7^ Low-dose LD has remained standard practice at the MDCS. In 1984, we published a 12-year experience. Dyskinesia and motor response fluctuations were between 10% and 20%^8^ compared with those reported^9^ on higher LD dose. We also found that dementia was not related to the duration of LD therapy^8^ (Figure 1).

At every MDCS patient visit, we used the newly described Webster^10^ motor symptom measurement scale and Global Disability Scale described by Hoehn and Yahr.^11^ When the Unified Parkinson’s Disease Rating Scale became available in 1987,^12^ we started using that. The differences between the older.^10^ ^,^ ^11^ and new^12^ scales were small and we could successfully convert old data to the new scale for publications.^13^

## What Should I Study and How Do I Do That?

In the late 1960s and early 1970s, two main topics of PD research were: (1) clinical observations on LD therapy and (2) biochemical studies of PD brains. I was just getting started when, in 1969, one New York group reported 100 patients treated with LD. Naturally, their data would be more credible than the observations I could report on a much smaller number of patients. Dr. Hornykiewicz pioneered the biochemistry in PD brains,^1^ which proved vital for future developments, but many more questions needed to be answered. We had neither the brain material nor the expertise for such studies.

I had to also consider the local realities. There was a major mismatch between my research interest and the institutional situations. There were no funds for clinician-driven research, no manpower dedicated to support research, no special equipment for such studies, and no culture of clinician-driven research at the University of Saskatchewan. We had no natural advantage to study movement disorders, based on population ethnicity or the occupation in the province. My job involved full-time teaching and clinical service with no protected time for research. Because I was unknown and from an unknown institution, I had to produce high-quality work that my peers would consider worthy of publication.

I realized that my research needed to progress beyond the clinical observations on the patients and had to settle on topics that were important but were not attractive to larger and faster research teams. I recognized that some such studies could be pursued in Saskatchewan. To do that, I needed the support of other experts; however, I had nothing special to offer to attract well-known collaborators. The research topics that I could tackle also required a long time to study. Because research was my choice, I had to find my own way to pursue it.

There was also a lingering doubt in my mind whether PD research was a good academic career option. In 1963, Poskanzer and Shwab.^14^ had reported that most PD cases were consequent to the von Economo encephalitis epidemic of 1917-1930s and postulated that when the population exposed to that epidemic passed away, PD would come to a natural end. It was published by a reputable group from a highly prestigious institution (Harvard), so we had to pay attention.

### Taking Advantage of the Local Situation and Building Alliances

My first major support came from my future wife, Karla. Before we got married, I told her that my salary was only $14,000 per year (the lowest for neurologists in Canada), but I could earn three times that amount in private practice. She asked me, “Do you like what you do?” I said yes. She responded, “Do not worry; we will make do”. She has done that for more than 40 years. Although she has been my longest and unwavering supporter—she worked in my laboratory as a volunteer for three years and as an employee for the next 15 years—her name does not appear on our papers.

The low salary was a mixed blessing because there was no pressure to generate large clinical billing income. The fees were (and still are) based on seeing a new or a return-visit patient and are significantly lower for a return visit. In my case, the fee structure did not matter and I decided to follow patients at intervals of my choice and spend as much time as I needed to perform clinical assessments that could be used for future research. When I could afford it, my first investment was a Super 8 movie camera. Anyone who could look through the lens and press the button took the video and every patient who consented had a movie made. Many patients had movies made before and on LD treatment. Soon after that, I purchased a Super 8 movie projector.

As the news of LD treatment for PD spread, invitations for lectures started to come and I never turned down an opportunity. The local media were very generous and showed the movies as part of public service. After a lecture to nurses’ alumni of the Saskatoon City Hospital, a lady said she wanted to work with me. I thanked her and told her that I had no money to pay her, to which she responded, “Did I ask you for money?” She was my first research assistant and her two sons, who were land surveyors, helped us free of charge to do a major study on the environmental cause of PD. Following a lecture to the Kinsmen’s Club of Saskatoon in 1972, I joined them for lunch. After the lunch, their executive members asked if they could borrow my film. They wanted to show the film to other provincial chapters to establish a foundation for the handicapped. The privacy regulations were not stringent at that time and without much thought I agreed to loan them the film. They established a highly successful annual charity event called TeleMiracle Saskatchewan. The first TeleMiracle was organized in 1977 with me and a dopa-responsive dystonia patient, whose film was used to motivate formation of the Foundation, as special guests. (My children, aged six and four, got their own thrills from meeting the *Sesame Street* actor, Bob McGrath, and even had their picture in the local newspaper.)

### Early Collaborating Scientists

My first effort to enlist scientific collaboration was to ask Dr. Bohdan Rozdilsky, the neuropathologist at the Royal University Hospital, to perform autopsies on my patients. He was trained by Dr. Olszewski (of Steele-Richardson-Olszewski fame), who was on our faculty in the 1950s. Dr. Rozdilsky was a thorough neuropathologist and a very nice man; together, we produced some 30 articles.

**Figure 2:**
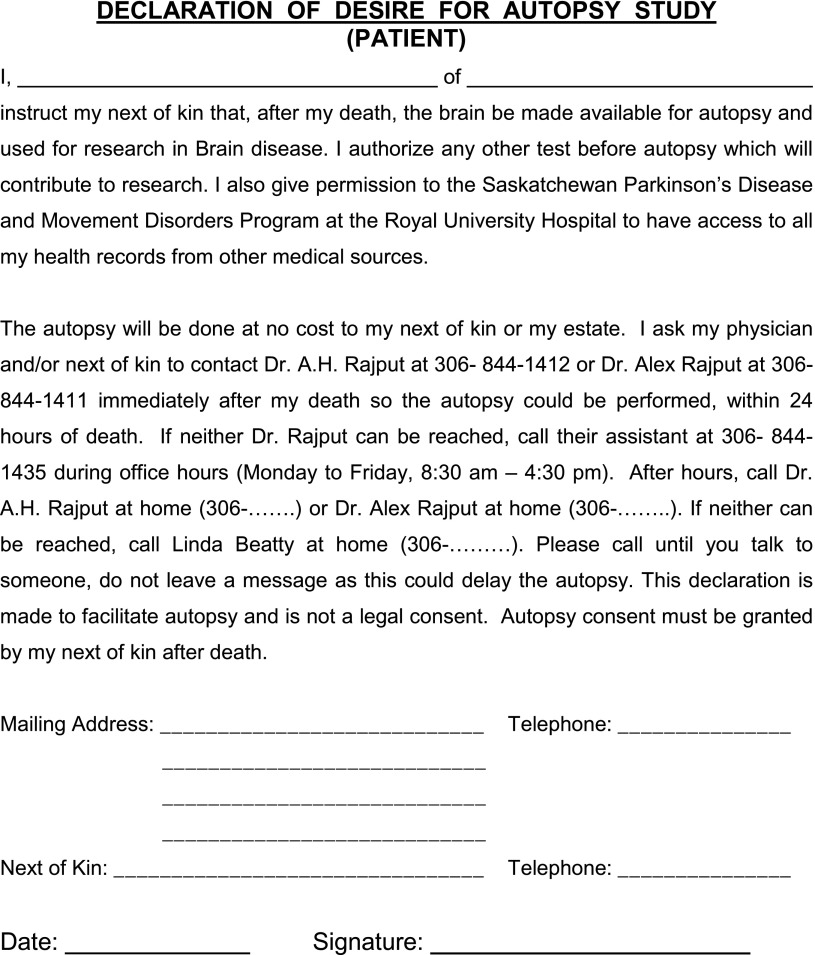
This form is provided to patients to decide on autopsy for research. A copy is provided to each family member and the family physician.

The first few autopsies were done on patients who had died in the hospital. Later, we decided to perform an autopsy on every patient if the family consented. That was a major undertaking in a large province of more than 652,000 square kilometers (251,700 square miles) but only one million population. We devised a plan such that patients/families would experience the least hardship. Patients interested in autopsy study for research would sign the Declaration of Desire for Autopsy Study form (Figure 2). A copy of the form was provided to each family member and to the family physician. The patients also signed a more detailed consent approved by the University of Saskatchewan Bio-Ethics Board allowing storage and use of brains for research. The final decision for autopsy was always made by the next-of-kin after death of the patient. Using local pathologists to perform brain autopsy was not suitable and frequently there was no local pathologist; therefore, it was decided to transport the body to Saskatoon for autopsy to ensure uniformity of the autopsy procedure. We concluded early on that for most of the movement disorders, one-half of the brain was sufficient for pathological studies and the other half could be frozen for future research. The autopsy had to be done within 24 hours of death to prevent major biochemical changes. The need for autopsy could arise at any time; therefore, I had to be available around the clock to arrange that. Initially, it took me a long time to get an autopsy organized, but the procedure has been streamlined—a typical autopsy now takes between 2 to 4 hours of neurologist’s time (though at times up to 12 hours).

### Outside Collaborators

We are a small institution and there was limited neurosciences expertise to study human brains. In 1967, Dr. O. Hornykiewicz had moved to Clarke Institute in Toronto, and by the mid-1970s we established collaboration with his team. Dr. S. Kish was his postdoctoral fellow and later a faculty member. We collected frozen half-brains in our freezer locally; when there was a sufficient number of specimens, they were shipped to Toronto.

I was also collecting cerebrospinal fluid and urine samples from PD patients untreated and on LD treatment. In 1981, my freezer was accidentally unplugged, resulting in the loss of more than 50 cerebrospinal fluid samples, several dozen urine samples, and about ten frozen half-brains—irreplaceable specimens. I was already a tenured full professor and research was not part of my job requirement. Devastated by the loss, I opted to discontinue this research. After three to six months of soul searching, I decided to give it one more try.

We made arrangements to have our freezers connected to the emergency power of the Royal University Hospital and to the hospital security alarm system. With the new arrangement, I had to be available 24/7 for any freezer mishap. There were several occasions when I had to come to the hospital at night or over the weekend to relocate frozen brains. After that, we started to keep all frozen half-brains in Saskatoon and sent brain samples to Toronto for specific research projects only.

By the mid-1990s, we had a sufficient number of frozen brains in Saskatoon to pursue several large studies. By then, Dr. Hornykiewicz had returned to Vienna. In 1996, while attending a meeting in Vienna, I asked Dr. Hornykiewicz to recommend someone who could help us process the frozen brains for research. He said, “Would you like me to come to Saskatoon and do it for you?” Before I could say yes, he said, “I know you do not have money; you do not have to pay me.” That was far more generous than I had expected. Soon he was appointed Distinguished Professor of Brain Disorders Research at the University of Saskatchewan with no salary or stipend. He came to Saskatoon once or twice a year for approximately a week at a time. He dissected the frozen brains and guided us in research. He could dissect brains for only three days per visit and approximately three new brains each day. Together with Dr. Hornykiewicz and his Toronto and new Vienna team, we have published more than 20 high-impact articles. Our first collaborative study was published in 1978.^15^ Dr. C. Pifl of the Vienna team is our new collaborator and together we have recently published a groundbreaking paper on the pathogenesis of PD.^16^

Dr. Alex Rajput joined the College of Medicine faculty in 2000. The next time Dr. Hornykiewicz came to Saskatoon, he spent the entire visit providing hands-on training to Alex. All the frozen brain dissections are now performed by Dr. Alex Rajput. With that development, our options to collaborate increased substantially.

## Sabbatical Leave

The cohort hypothesis^14^ (of PD and its relation to von Economo encephalitis infection) was stuck in my mind. When I could not convince anyone else to study that, I took a one-year sabbatical leave (1980-1981) to study the epidemiology of PD in Rochester, MN. They had previously reported two incidence studies from 1935 to 1966. If the cohort hypothesis were correct,^14^ it was expected that the incidence of PD would start to decline by the late 1960s. Using the same methodology as in the previous studies, I decided to study the incidence of PD in Rochester between 1967 and 1979. Our study^17^ showed no decline in the incidence of PS; thus, the cohort hypothesis^14^ was put to rest.

The sabbatical leave experience was wonderful academically but financially disastrous. My salary was reduced by 35% and we had to maintain two residences—Saskatoon and in Rochester for part of the year. The Canadian dollar went down to 65 cents and interest rates climbed to more than 20%. My host and mentor, Dr. Len Kurland, was very generous to me and our family. Mayo also helped me analyze my own clinical data, which by now were voluminous.

I pursued an analytic epidemiology study to identify environmental cause(s) of PD. It was like looking for a needle in a haystack—because clinical onset of PD is later in life, one has to consider numerous environmental factors. I decided on having a smaller haystack for the search. We included only those patients that had PD onset by age 40 years and further restricted the environmental consideration to the first 15 years of life (because at age 16 children can leave home). Twenty-one early-onset PD patients in Saskatchewan were identified; all except one were born and raised in small communities or on a farm and they had all consumed well water for the first 15 years of life.^18^ We decided to analyze the well water from those locations. Most old wells were not operational, so we identified the nearest functioning wells. Water samples were collected and compared with Saskatoon tap water—there was no difference.^19^ We sent those water samples to Dr. W. Langston in California for possible 1- methyl -4- phenyl -1,2,3,6-tetrahydro pyridine (MPTP) or related substance, but none was identified (unpublished).

Saskatchewan is a major agricultural province where herbicides and pesticides have been used for as long as they have been commercially available in Canada. We did not find an association between the use of any of the herbicides or pesticides and higher incidence of early onset PD.^20^

Considering my limited resources, I did not pursue population epidemiology further.

## The Second Phase of Research

The two phases of our research are arbitrary because there is much overlap. The dividing line for me was excluding the cohort hypothesis; I could now focus on clinical and pathological studies of movement disorder brains.

By contemporary standards, we had devised a new model of research that had a distinct set of needs. This model was dictated to a considerable extent by the local circumstances. We had major weaknesses, but there were several strengths as well. There was a need for research support at multiple levels, especially because our research was based on human subjects. The major clinical focus was longitudinal follow-up of the MDCS patients and autopsy studies of their brains. That required strong patient/family/public involvement. Saskatchewan was the home of the cooperative movement that started during the Depression years. In 1962, Saskatchewan became the first Canadian province to introduce general tax-funded universal health care system, and I wanted to capitalize on that public spirit. Personally, I needed to go beyond the usual duties of a physician—to lead by example. Building the trust and support of the public required different skills than the practice of medicine; we have succeeded at that and much credit goes to the people of Saskatchewan.

### Funding

Funding for clinician-driven movement disorders research in Canada is mainly from local private sources. The researchers procure funds with their own efforts. A lot of our work was done on “please and thank you,” much personal unpaid time, and significant in-kind support from the hospital. In spite of that, we still needed funds to pay our small dedicated research staff, and for equipment and consumables. Saskatchewan does not have many wealthy people who could make large donations. Some patients/families offered small amounts of money for special clinics and research. I was not sure how to handle those funds and consulted a senior colleague; he recommended against personally handling any donations. Four of my patients/friends and I applied for a registered charity status and in 1972 we received approval for the Saskatchewan Parkinson’s Disease Foundation (SPDF). The primary stated objective in the application was to raise funds for special clinics and research. Anyone who wanted to donate towards my work was directed to the SPDF. The donations were usually small. I observed early on that I would prefer receiving one dollar from a million people as opposed to a million dollars from one person (million-dollar single donations were not forthcoming anyway). The donors of one dollar I hoped would be able to give another dollar the following year. The large number of small donors also started to identify with our program and continued to support it in many other ways, notably in getting the brain autopsies. The SPDF contributed financially to the running of special clinics and research, from those donations. The SPDF was subsequently renamed as Parkinson’s Society Saskatchewan.

In the early 1990s, a group of patients/families organized an annual curling tournament in Regina to support our clinics and research. Soon, another group organized a golf tournament in Regina with the same objectives. Those two events have been held regularly for more than 22 years and have raised millions of dollars for the program. Both are strictly volunteer-run events. Some other smaller provincial groups have organized independent fundraising events to support our work. We have received money from multicenter drug trials (including some funded by the National Institutes of Health in the United States); all funds left over from the drug studies were kept for other research and the special clinics. Even after I (Ali) retired from my position at the University of Saskatchewan in 2002 (when mandatory retirement at the University of Saskatchewan was still in place), I did not take residual research funds for myself. We have received research grants from Parkinson’s Society Canada and small amounts from the International Essential Tremor Foundation and from Canadian Institutes of Health Research joint grants. In 2008, an anonymous donor established a one million dollar Trust for the Movement Disorders Research and later donated $600,000 more. Our model of program funding is similar to that at other major movement disorders programs in Canada. Since my (Ali) retirement from the University, the provincial government has provided financial support towards Movement Disorder Clinics.

## Additional Collaborations

After Dr. Alex Rajput was trained in processing the frozen brains in 2000, the number of collaborators has increased. So far, we have had 17 different collaborating teams from Canada, the United States, Europe, and Japan. We will not go over the contributions made by all of them and instead will restrict our comments to two major Canadian teams that are currently active.

### Quebec City – Laval University Group

In 1997, Dr. Paul Bédard, Professor of Neurology, Laval University, Quebec City, visited Saskatoon for the Canadian Congress of Neurological Sciences. Paul was pursuing high-quality work on a primate model of MPTP-induced Parkinson syndrome (PS). The MPTP model has served well for studies of major motor features of PS, but does not fully represent the naturally occurring disease.^16^ I asked Paul to consider enlarging his scope of work to include studies of human movement disorder brains and later that year I invited him as a Visiting Professor to the University of Saskatchewan. We showed him our setup and he was convinced that we could develop a useful collaboration. He recommended inclusion of his younger colleague, Dr. Thérèse Di Paolo, who had done considerable work on MPTP monkeys. She visited Saskatoon the following year and our collaboration began. She introduced us to her younger colleague, Dr. Frédéric Calon. All three of them have honorary Adjunct Professor appointments at the University of Saskatchewan. This collaboration has produced more than ten very high-quality papers. They have studied PD brains including LD-induced dyskinesia.^21^ Our recent paper in *Brain* ^22^ on the GABAergic system in ET brains was chosen as one of the ten best scientific developments in the Province of Quebec in 2012. We should note that Quebec is a powerhouse for neurosciences research in Canada. Our collaboration continues with Drs. Calon and Di Paolo, but unfortunately Dr. Bédard is no longer active.

## Mayo Clinic Jacksonville, Fl/University of British Columbia

In 2002, I (Ali) was introduced to Dr. Matthew Farrer who was pursuing genetic studies of PD at the Mayo Clinic, Jacksonville, FL. We invited him to Saskatoon and quickly realized that we would benefit from each other’s expertise. Dr. Farrer and his team, including Dr. C. Vilariño-Gűell, moved to the University of British Columbia in 2010. Our University of British Columbia collaboration team has enlarged and now includes two leading movement disorders and positron emission tomography scan experts, Drs. Jon Stoessl and Silke Cresswell. Together, we have published more than 25 articles dealing with the genetics of movement disorders and more recently with functional imaging (positron emission tomography). We have reported on the *LRRK2* gene mutation with autopsy verification in one PD family; interestingly, the pathology showed a tauopathy with no evidence of Lewy bodies.^23^ Dr. Farrer and his group have identified *DNAJC13* mutation in a large multi-incident Mennonite family from Saskatchewan, with autopsy verification of Lewy body PD.^24^ This observation has the potential to significantly enhance our understanding of the pathogenesis of PD. There are several other articles accepted or in the pipeline.

## Students and Future Scientists

As the research in movement disorders at the University of Saskatchewan progressed, several students worked with me during the summers. Even students joining our program for a short time have been able to present and publish articles based on our resources. Some of them now occupy prominent academic positions in Canada and the United States. Notable in that group are Drs. Ryan J. Uitti and Alex Rajput. Ryan started working with me in the summer after his first year of medical college and then continued to be involved throughout his medical college years and beyond. He graduated in 1988 and is now Professor of Neurology at the Mayo Clinic, Jacksonville, FL. Dr. Alex Rajput started working with me in the summer when he was 15 years old and I paid him from my own pocket. My motive was more personal than academic—I wanted to know where my son was so he would not be bored and get into trouble. He did his neurology training at the University of Iowa and moved back to Saskatoon in 1998 as he was on a J1 visa in the United States. He needed a postgraduate year-5 of residency training to qualify for the FRCP(C) Neurology Examination and following that he did a one year movement disorder fellowship in Saskatoon. He had an offer for a position at the University of Saskatchewan but was seriously considering moving back to Iowa. I did not want to get involved in that decision nor did I participate in his appointment to the faculty at the University of Saskatchewan. Once he was here, I asked Dr. Hornykiewicz to train him in the methods of frozen brain dissection in 2000. Alex also did special training in 2003 on the rotenone rat model of PD at Emory University, Atlanta, in Dr. J. Timothy Greenamyre’s laboratory. In our laboratory, the mortality in those animals was high and we determined that early death was associated with brain hemorrhage.^25^ We concluded that this model was not suitable for our research. Dr. Alex Rajput is now the Director of the Saskatchewan Movement Disorders Program, including the clinics, research, and all related laboratories.

## Main Components of Saskatchewan Movement Disorders Program

There are two inseparably linked components: (1) clinical services for Saskatchewan patients and (2) research. That dual objective was the foundation of this program in 1968. The data collected in the clinics are not driven by any one research protocol but are research-worthy. The clinical data are regularly used in conjunction with videos, pathology, biochemistry, and genetics information in our research. Longitudinal follow-up of the patients is a major feature of the MDCS. All Saskatchewan residents carry general tax-supported health care insurance and access to MDCS is equal to all provincial residents. There is a provincial plan to support drug costs.

### Clinical Data

Every patient seen at the MDCS is regarded as a potential candidate for inclusion in research studies. The clinics in Saskatoon and Regina are identical. At every clinic, there are two or three support staff members assisting the clinicians. Video recordings are made on all consenting subjects at and rarely on consenting family members who may be of research interest. After initial MDCS assessment, a brochure outlining the disorder, the nature of the disease, and management options is provided. The expected outcome is discussed with the patient/family. Those discussions are not restricted by time. Patients are provided with our office telephone numbers, and have an unlimited access to the two (AHR/AR) neurologists between clinic visits. We (AHR, AR) answer our patients’ telephone calls, and considerable neurologist time is spent on the telephone with patients. We are interested in knowing of changes in the patient’s status; when appropriate, we initiate treatment changes that may include phone calls or faxes to the patient’s pharmacy—that information is entered into the patient record and used for patient care and research.

At an opportune time, we ask patients to consider if they wish to have an autopsy be done after death (Figure 2). We prefer to give that form to the patient/family to take home and discuss with family members before signing. If the patient is comfortable and intends to proceed with the autopsy, the form is signed and returned to us. There is no expense to the family and the body is not disfigured for viewing. They are also assured that regardless of the decision on autopsy, there would be no impact on the ongoing care. In case the patient is not comfortable with the declaration,he or she is asked to write “no” on the form and return it to us. Once a patient has said “no,” we do not ask again for autopsy declaration. In those cases where the patient has declared the desire to have an autopsy, a copy of the signed form is provided to each family member and to the family physician. Patients that sign the declaration also sign consent for use of brain tissue for research and allow us access to their clinical information from all sources. If we come across a suitable normal control subject, we offer a similar autopsy option.

## Autopsy Procedure

Typically a call comes to the neurologist from a family member, nursing home, or hospital personnel that the patient has died and the family wishes to have an autopsy performed for research. The two of us (AHR, AR) are on 24/7 call (Figure 2). We make autopsy arrangements with the family, care home, funeral home, morgue attendant, etc. and are available from the time of initial call until the body is released back to the funeral home. Autopsies are performed in Saskatoon. The cost of transporting the body to and from Saskatoon is our responsibility. Saskatchewan is a large geographical area so there can be long distances to transport the body. We also pay for the time of extra morgue attendants when they are needed after hours. The average immediate cost for body transport and the autopsy procedure is now approximately $2,000. In rare situations, for patients that have died out of province we are grateful to have received support from local neuropathologists.

Between 30% and 35% of our Parkinson’s patients and a smaller percentage of ET cases come to autopsy. The decision to have an autopsy performed rests with the next-of-kin; there are rare examples in which the patient did not sign the declaration but the family decided on autopsy. On other occasions, patients not seen at MDCS also come to autopsy. A call would come at night indicating that someone in a nursing home who had PD has died, but neither the caller nor the neurologist is certain if the patient was ever evaluated at the MDCS. The autopsy will proceed and when we discover later that the patient was never seen in our clinic, the brain is not used for research because of lack of our own clinical information. Some normal controls not seen at the MDCS also come to autopsy.

Immediately after removal, the brain is divided at midline into two halves. Half is frozen at −80°C for future studies and the other half is fixed in formalin for pathology studies. (There is no particular side that is chosen when the brain is grossly normal. For those cases in which there is a known structural lesion, that hemisphere is fixed in formalin.) The neuropathologist produces a detailed report that is attached to the patient’s clinical record and a copy is sent to the next-of-kin, with an offer to discuss the diagnosis.

**Figure 3:**
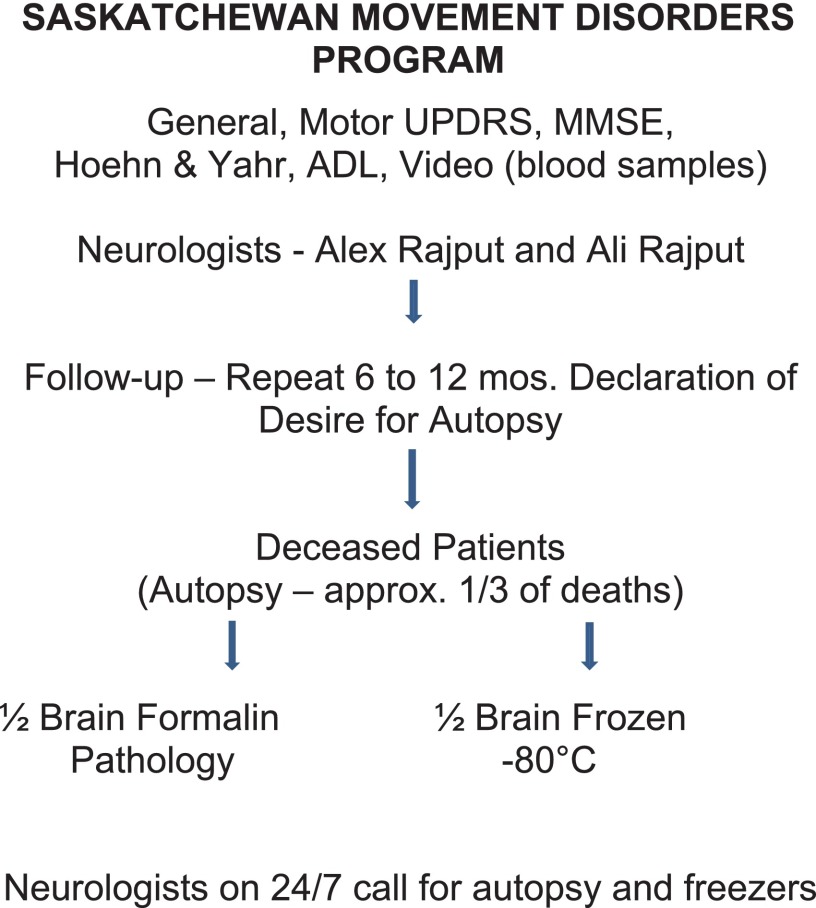
Flow chart of Saskatchewan Movement Disorders Program operations.

Figure 3 shows summary of Saskatchewan Movement Disorders Program.

## Information and Material Storage

**Figure 4:**
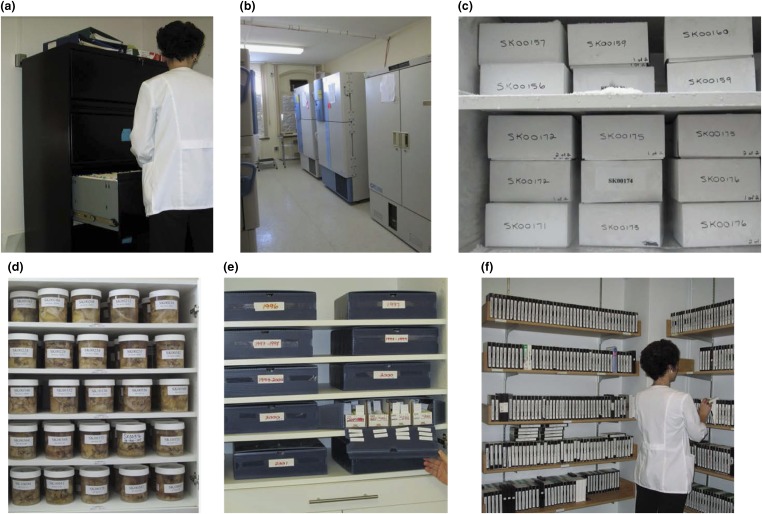
Pictures of Saskatchewan Movement Disorders program storage of patient records and research material. (A) Filing cabinet containing hard copies of patient clinical records. (B) −80°C freezers. Currently there are nine freezers. (C) Cardboard boxes, each containing half-frozen brain from a patient. Each box has patient identification at four places—two with only the number and two with name and number. (D) Formalin-fixed remains of the brain tissue after pathology has been completed. (E) Paraffin blocks and glass slides stored in our laboratory. (F) Video library.

Figure 4 shows different laboratories where patient records and material are stored.

Videos made with older technology need updating. There are more than 3500 patient videos and some patients have had several videos. One member of our staff is responsible for the video library. The videos are updated regularly to ensure compatibility with the most recent technology and catalogued.

As noted previously, the freezers are connected to the hospital auxiliary power and the alarm system monitored by Royal University Hospital security department. The two of us (AHR, AR) are on 24/7 call for any freezer mishap.

Each frozen brain is kept in a separate box. The frozen brains are dissected (by AR) in another special room and only one brain is brought to that room at a time.

The formalin-fixed tissue and paraffin blocks are being used for research with increasing frequency as new investigative tools become available. One member of our staff has the primary responsibility for the brain tissue storage laboratories, though other members are familiar with that.

### Major Movement Disorders Studied at MDCS

**Figure 5:**
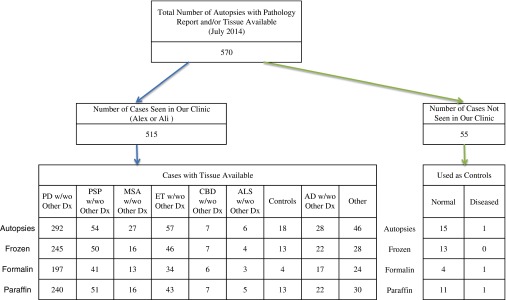
Summary of autopsy studies performed at Saskatchewan Movement Disorders Program and available samples. PD=Parkinson disease; PSP=progressive supranuclear palsy; MSA=multiple system atrophy; ET=essential tremor; CBD=corticobasal degeneration; ALS=amyotrophic lateral sclerosis; AD=Alzheimer disease.

The disorders studied at MDCS are those prevalent in the Saskatchewan population. The three most common disorders in our clinic population are parkinsonism, ET, and dystonia (mostly focal or segmental). Figure 5 shows the latest autopsy count and the broad diagnostic groups. In some cases, a final pathology report was not available at the time of this article’s preparation. Most of our collaborations are based on deidentified frozen half-brains. Numbered brain tissue samples are provided to the collaborators.

## Our Resource Is Not a Brain Bank

Since we started publishing human brain studies, interest in this field has increased. Several institutions are now pursuing studies of movement disorder–autopsied brains. Each program uses the strategy appropriate to the local circumstances and each has its own strengths and weaknesses. Following are the common models of such programs.

1. Patients receiving a certain drug treatment regimen are enrolled in research; thus, they are evaluated by the researchers at regular intervals using specific research protocol and autopsy studies are performed.^26^ The patients, however, are cared for by their own physicians. The strength of such programs is that research-specific data are collected prospectively. The weakness is that only the specially selected cases are included and the clinical data collected by treating physicians are heterogenous.
2. Studies based on individuals from defined communities who express a desire to have autopsy studies for research.^27^ ^-^ ^30^ Strengths of such programs are that patients are periodically evaluated in detail by movement disorders experts. They can collect large amounts of detailed data and a large number of autopsied brains, including control brains for research. The autopsy can be obtained soon after death and expert pathologists study every brain. The weaknesses are that the cases included are all elderly and not representative of the general population.^29^ ^,^ ^30^ and in many cases the medical care is provided by other physicians.
3. National Parkinson’s Disease Brain Bank.^31^ Brains from all parts of the country are forwarded to a centralized brain bank. The strength is that a large number of brains can be collected. The weaknesses are that there are many (more than 70) physicians including neurologists and geriatricians who look after these patients and collect the clinical data.^32^ The autopsies are performed locally at multiple sites, thus there is a lack of uniformity of the clinical data and the brain harvesting.
4. National Essential Tremor repository in the United States.^33^ ^-^ ^35^ Neurologists—mostly movement disorders experts from across the country—provide the brains from their own patients. The strengths are that a large number of ET brains are available for research and there is very good team of neuropathologists that study those brains. The weaknesses are that the patients are looked after by many different neurologists and the autopsies are performed at multiple sites before the tissue is transported to the central repository.
5. Our program is based on our own clinical practice of movement disorders. For research, we include patients that we (AHR, AR) have evaluated at the MDCS. Our cases include a representative sample of the provincial population—all ages and all forms of treatment. The autopsies are done at one centralized location. All patients are looked after by us (AHR, AR), which ensures consistency of the clinical data. The treatment regimen is fully documented and the autopsy procedure is standardized. All patient records including videos and brain material are preserved in our laboratories. Research is based on movement disorder patients seen in our clinic and controls. The main weaknesses are that it is a slow process to collect large number of brains and the clinical data are not based on any specific research protocol. Over the years, we have collected several hundred brains from patients with diverse movement disorders (Figure 5) and the clinical data are of adequate quality to complement other major research studies.^8^ ^,^ ^13^ ^,^ ^36^ ^-^ ^38^

After visiting Saskatoon, Dr. Andres Lozano, Professor and Head of Neurosurgery, University of Toronto, called it a “Safety deposit—world heritage site unmatched anywhere.” Dr. Hornykiewicz observed, “I consider that collection the most valuable in the world. There is nothing else like it, and I know about every such lab. He collected the whole thing in Saskatoon very patiently, and now it is unique. He has very rare material and has meticulous records of the patients.” To distinguish from a brain bank, we call our resource the Brain Safety Deposit.

## Selected Work of the Saskatchewan Movement Disorders Program

For space reasons, we cannot comment on most of our articles and will exclude the epidemiological studies noted previously. We briefly describe some of our work in chronological order to illustrate the evolution of the research over time.

1. 1973. *Specificity of Tremorilytic Effects of Alcohol and Propranolol*.^4^ Improvement of ET after an alcoholic drink has been anecdotally known for a long time and that information was passed by the neurologists from one generation to the next, but it was never established scientifically. This was first ever literature report on systematically studied effect of oral alcohol on the ET.
2. 1975. *Relative efficacy of alcohol and propranolol in action tremor*.^5^ This was the first published study on the effect of 1 oz (30 mL) oral alcoholic on the action tremor in several different disorders. We reported on 39 cases, including 21 with ET and 15 with PD. Action tremor improved 30 minutes after 1 oz of alcohol ingestion in 62% of ET and 47% of PD cases; action tremor of some other rare disorders also improved. The blood alcohol levels did not correlate with the tremor benefit. More patients improved on propranolol than with a single alcoholic drink. To our knowledge, this is the only study on the effect of oral alcohol in action tremor in multiple disorders. The symptomatic benefit of alcohol on action tremor is neither specific nor restricted to ET.
3. 1976. *Dysautonomia in parkinsonism: a clinicopathological study*.^7^ Autonomic dysfunction in PD was recognized by early 1960s, but the anatomical basis of that was unknown. Most pathology studies were limited to brain and the spinal cord. This study included eight patients that had detailed intra-arterial blood pressure assessments in supine and upright positions, and the autopsy study included the brain, spinal cord, and the sympathetic ganglia. The orthostatic hypotension correlated with the sympathetic ganglia pathology—neuronal loss and Lewy body inclusions. This is the only study to our knowledge that looked histologically at central as well as the peripheral autonomic nervous system for the autonomic dysfunction in PD.
4. 1978. *Receptor basis for dopaminergic supersensitivity in Parkinson’s disease*.^15^ This included 14 normal controls, 6 untreated PD, and 5 LD-treated PD patient brains. The dopamine receptors were supersensitive in the untreated PD and the sensitivity declined after treatment with LD. This is one of the earliest studies, if not the earliest study of its kind.
5. 1982. *Reversible drug-induced parkinsonism*.^39^ Drug induced parkinsonism has been well known since the 1950s, but there were no pathological studies to determine the underlying mechanisms of parkinsonian manifestation. This was a clinical and pathological study of two cases. On neuroleptics, each patient manifested parkinsonism. At autopsy, each case had pathological evidence of premotor PD. The neuroleptic stress needed to unmask parkinsonism was inversely related to the underlying PD pathology.
6. 1984. *Chronic low dose levodopa therapy* .^8^ High dose of LD was a common practice in the 1960s-1970’s. We observed that this resulted in early onset of dyskinesias in many cases and decided to use a lower dose of LD. This was a 12-year clinical study of 195 cases on low dose—3 g or less plain (600 mg LD/carbidopa)/day. Low-dose LD was effective and resulted in considerably lower incidence of dyskinesia and motor response fluctuations.^8^ ^,^ ^40^ compared with most literature reports.^9^ Those observations were confirmed 29 years later by Olanow et al.^41^ Our practice of using lower dose resulted in lower drug costs, less common need for medical service for LD complications, and a better quality of life for patients.
7. 1990. *Levodopa efficacy and pathological basis of Parkinson syndrome*.^36^ LD was known to be effective in most PD cases, but the information was based on clinical observations. Some patients in those studies likely included other variants of PS. This is a 22-year clinicopathological study of 59 cases. It shows that all PD patients receiving an adequate LD dose improve, all patients with Substantia Nigra (SN) restricted pathology also improve, and 33% of multiple system atrophy cases have some benefit on LD. This is the first published autopsy verified report on LD efficacy in PS.
8. 1991. *Accuracy of clinical diagnosis in parkinsonism—a prospective study*.^42^ This was the first study comparing clinical diagnosis with autopsy verification in parkinsonism. At the first visit, a neurologist correctly diagnosed PD in 65%, whereas at the final assessment before death, diagnosis by a movement disorder neurologist was accurate in 76% (as verified by brain histological studies). This article was very well-received and was considered the best original article in the *Canadian Journal of Neurological Sciences* in 1991. In 2014, Adler et al^28^ reported a virtually identical accuracy rate*—*68% of the initial and 77% of the final clinical diagnosis accuracy. Thus, predicting the pathology, based on the clinical diagnosis of PD, has remained unchanged for more than two decades.^43^ Our study helped include clinical diagnostic inaccuracy when considering PD case selection for drug trials.
9. 1993. *Significance of parkinsonian manifestations in essential tremor*.^37^ Tremor is the major manifestation in both PD and ET, but resting tremor is considered characteristic of PD. There is no widely available biological marker to clinically distinguish between those two disorders. In this clinicopathological study of nine ET patients, three (33%) had resting tremor as part of the natural evolution of ET. This was first pathologically confirmed report on the presence of resting tremor in ET patients. We also recommended that in a well-established ET case, all three parkinsonism motor features*—*resting tremor, bradykinesia, and rigidity (preferably asymmetrical)*—*must be present before making the additional diagnosis of PS.
10. 1993. *Prognostic significance of the onset mode in parkinsonism*.^44^ It was widely known that parkinsonian cases have different modes of motor onset, but the reasons for that were not known. This clinicopathological study of 70 autopsied patients over 29 years showed that most patients with tremor onset had PD, whereas the postural instability and gait difficulty onset was most common in multiple system atrophy and progressive supranuclear palsy. The prognosis is most favorable in the tremor onset cases.
11. 1994. *Dopa-responsive dystonia: pathological and biochemical observations in a case*.^45^ Dramatic and sustained response on a small dose of LD is well-known in dopa-responsive dystonia. This was a detailed clinical, pathological, and brain biochemical study of a 19-year-old woman who died after 14 years of dystonia symptoms. She had marked improvement on LD initiated at age 8 years and died in an automobile accident at age 19. The number of pigmented substantia nigra neurons was normal, but there was a marked reduction in the neuronal pigmentation. There was marked reduction in striatal dopamine levels. This patient was subsequently reported in a genetic study showing *GCH1* gene mutation as the first example in a Caucasian subject.^46^ Her brother, who had mild symptoms from childhood but did not come to medical attention until age 49 years with mild features of parkinsonism and dystonia, has responded very well to a small dose of LD.
12. 1997. *Is levodopa toxic to human substantia nigra?*.^47^ Toxicity of levodopa to the human SN was vigorously debated based on laboratory studies, especially after dopamine agonist drugs became available. We reported five cases including two autopsies. In one patient, a total of 24 kg (plain LD) was used over 26 years without evidence of nigral damage. This was the first pathologically verified study to show that levodopa is not toxic to human SN. We have now collected 21 LD-treated autopsied cases that have normal SN (unpublished).
13. 1997. *Timely levodopa (LD) administration prolongs survival in Parkinson’s disease*.^48^ There was an ongoing debate if LD increased life expectancy in PD; however, it could not be verified because all the contemporary PD patients are treated with LD and untreated controls needed for comparison are not available. This clinical study included 934 cases observed over 22 years. Cases were divided into those with delayed access to LD at first assessment at MDCS (1968 to the end of 1973) with those having unrestricted access to LD (if needed) at first assessment (1974 and later). To our knowledge, this is the only study comparing PD patients that had highly restricted access to LD with patients that had unrestricted LD access. The survival in parkinsonism remains shorter than expected, but it has increased significantly since unrestricted access to LD. Survival benefit, however, is restricted to those that receive LD therapy before onset of postural instability (stage 3 Hoehn and Yahr, or modified Hoehn and Yahr stage 2.5). We also noted that epidemiological studies comparing PD survival with the general population that start from the retrospectively identified PD onset date artificially inflate PD survival. Such survival comparisons should start from the date of PD patient entry in the study (i.e. first visit).
14. 2002. *Clinical-pathological study of levodopa complications*.^38^ LD motor complications are known to increase with longer duration of treatment; however, most studies are based on clinical observations of heterogenic PS cases that have different responses to LD.^36^ This was a 28-year study of 42 autopsied Lewy body PD cases. The most common LD motor complication was dyskinesia. Cumulative incidence of dyskinesia (observed at some time during the course) over the 20-year study interval was 80%, of wearing-off 50% and of on-off 25%. To our knowledge, this is the only study based on autopsy-verified PD cases on this subject.
15. 2004. *Human brain dopamine metabolism in levodopa-induced dyskinesia and wearing-off*.^49^ Some PD patients on long-term LD treatment develop wearing off but no dyskinesia, whereas others on comparable LD dose/duration manifest dyskinesia but no wearing off. The reason for that difference was not known. This study compared nine PD brains of cases with different LD motor complication profiles followed over 21 years with four neurologically normal control brains. This is the first autopsy study to show that patients with wearing-off metabolize dopamine more rapidly than the patients with dyskinesia.
16. 2004. *Essential tremor course and disability: a clinicopathological study of 20 cases*.^50^ ET patients have a variable course—in some cases there is no significant progression and in others the symptoms worsen with time. In 20 autopsied ET cases seen at MDCS over 32 years, we observed progressively wider anatomical sites of tremor involvement and increasing functional disability with time. The risk of developing PD in the ET cases was comparable to that in the general population. This is the first autopsy study of longitudinally followed autopsied ET cases.
17. 2008. *Globus pallidus dopamine and Parkinson motor subtypes: clinical and brain biochemical correlation*.^51^ Some PD patients manifest dominant tremor, whereas others have dominant bradykinesia and rigidity, and many more have equal severity of those motor features. There was no explanation for those clinical differences. This study of eight PD cases that had different motor subtypes (determined when the entire clinical course of disease was considered) and five normal control brains found striatal dopamine loss was more pronounced in the akinetic-rigid compared with tremor dominant cases. In the globus pallidus interna, dopamine loss was the most pronounced in the akinetic-rigid and the least in the tremor-dominant cases. These data indicate that akinetic-rigid cases have a more widespread pathology than the tremor dominant cases.
18. 2009. *Course in Parkinson’s disease subtypes: a 39-year clinicopathological study*.^13^ Having established that the akinetic-rigid cases have more advanced pathology,^51^ we chose to determine the course of disease in different PD motor subtypes. This study included 166 autopsied PD cases seen at MDCS during 39 years. Motor subtypes were based on the entire course of disease. The outcome was most favorable in those who had tremor-dominant and the worst in akinetic rigid cases. This is the longest followed autopsy-verified study of PD motor subtypes reported to date.
19. 2011. *Significance of cerebellar Purkinje cell loss to pathogenesis of essential tremor*.^52^ The pathological basis of ET remains unknown but some studies.^33^ ^-^ ^35^ reported that cerebellar Purkinje cell (PC) loss was the specific pathology in a large majority of ET cases. This study of seven ET, six tremor-dominant PD and two normal control brains, found no difference in PC counts between ET, PD, and normal controls. Literature evidence supporting that PC loss is not specific to ET and the conclusion was discussed. Because some criticized the study due to small numbers, in 2012 we reported on 59 autopsy cases including 12 ET, 41 PD, and 6 normal control brains.^53^ The PC counts were done by a neuropathologist blinded to the clinical diagnosis. It revealed that the PC counts were marginally higher in the ET cases compared with the control groups. A recent large, independent study has confirmed our observations.^54^ It is concluded that PC loss is not the pathological basis of ET.
20. 2012. *Defective dentate nucleus GABA receptors in essential tremor*.^22^ This study of 10 ET, 10 PD, and 17 control brains first time showed reduced GABA receptors in the dentate nucleus in ET cases.
21. 2014. *DNAJC13 mutation in Parkinson disease*.^24^ This study reports on a large multi-incident Mennonite family with three autopsy-confirmed Lewy body PD that had DNAJC13 mutation. The first case was seen nearly 30 years before publication, but took this long to evaluate other family members and for some affected persons to come to autopsy. Other seemingly unrelated persons from four different families of Mennonite ancestry were identified; all but one family can trace their roots to the Chortitza Mennonite colony of the Ukraine. The DNAJC13 mutation leads to toxic gain of function resulting in impaired endosome transport. This observation furthers our understanding of the pathogenesis of PD.
22. 2014. *Is Parkinson’s disease a vesicular dopamine storage disorder? Evidence from a study in isolated synaptic vesicles of human and nonhuman primate striatum*.^16^ The molecular mechanism of onset and progression of PD pathology remains unknown. This study included six PD, four normal control brains, and seven MPTP treated and eight normal control monkey brains. There was markedly reduced dopamine vesicular uptake and binding in the human PD, but not in the MPTP-treated monkey brains. This abnormality is therefore specific to PD. This observation improves our understanding of the pathophysiology of PD and reinforces that despite the usefulness of the MPTP model of PD, it does not represent fully what is going on in human PD.

## What Made Our Research Model Successful?

Compared with most other centers, we did not have any special advantages of funding, equipment, manpower, or technical skills. We believe the success of our research program can be attributed to the following.

1. Fully integrated clinical and research programs since it was started in 1968. A large number of cases can be studied because the program includes all movement disorders seen at the MDCS. We follow the patients longitudinally, make videos on all consenting subjects, and perform autopsies wherever possible. All patients are evaluated at each visit by the same two neurologists, ensuring consistency of the clinical data.
2. A realistic plan, taking into account the local circumstances.
3. Building a long-term sustainable research program. It has several components and each involves successful integration of multiple individuals.
4. Slow but patient. In the earlier years, many of my (Ali) contemporaries “appeared to be flying in supersonic jets, while I was walking.”
5. Hard work. In the earlier years, I (Ali) took virtually no holidays and the research was done over the weekends and evenings. Although there are many other examples of extraordinary efforts by Saskatchewan Movement Disorders neurologists, one that is easy to understand is the availability. One of us (AHR) has been on 24/7 autopsy call for 46 years and emergency freezer call for 32 years, whereas the other (AR) has been on each of those calls for 14 years, without financial or academic reward.
6. The support of many others, including the hospital personnel, who have contributed significantly to this program in their own ways.
7. Saskatchewan public support. Saskatchewan people are probably the most generous anywhere, when the term “generosity” is used in a broad sense. Not only has the public raised large sums of money from many small donations to support this work, they have also helped collect research-worthy data and many families authorized autopsy on their loved ones—even when there was no possibility of any gain to the family.
8. Cost-effectiveness. Not counting the many voluntary functions performed by thousands of Saskatchewan people and neurologists over the 46 years, this program has so far cost more than $20 million. The cost was bearable as it was spread over a long period.

There is an African saying: “If you want to go quickly, go alone; if you want to go far, go together.” Together, the Saskatchewan public, neurologists, and our collaborators have indeed traveled far—starting from nothing to being what is considered by some experts in the field as the best program of its kind.

## Future

Our efforts were dictated by circumstances, and circumstances change with time. The future of this program depends on the new local realities—the desire of the new local expert neurologists in this field and the institution to pursue this model of research. If handled properly, the future of the program is bright. We have one of the best resources and have the mechanism in place for ongoing replenishment. This program can expand to include many other aspects of PD, ET, and dystonia and also include other movement disorders.

## Foreseeable Needs

We are not likely to receive a multimillion dollar donation or to have a very large neurosciences manpower or technological superiority over other major centers. This research program is anchored to patient care and specialized research-worthy clinical data collection. Those functions are performed by the specialty trained neurologists. By the standards of this institution, it is a fairly large program. The demands on our (AHR, AR) time are numerous and we are frequently the bottleneck for the collaborative studies. We appreciate the patience of our colleagues. The program has reached a point that the two of us, with one (AHR) retired from the university (though spending more than 50% of his time on research), cannot take full advantage of the available opportunities. There are many research options we cannot pursue because of a lack of medical manpower. There is a need for three fulltime academic movement disorder neurologists if we wish to realize the full potential of this program. Appropriate recognition and support for the multiple tasks which neurologists perform is vital in order to attract and retain movement disorders experts.

Those who can contribute locally with specialized clinical or imaging assessments as well as basic science research support are also needed. New collaborators that bring state-of-the-art expertise to take advantage of our unique resource are welcome. We are highly optimistic about the continuing public generosity; however, there is a need for a more sustained and reliable funding source.
